# Internet Addiction Test (IAT): Which is the Best Factorial Solution?

**DOI:** 10.2196/jmir.2935

**Published:** 2013-10-09

**Authors:** Palmira Faraci, Giuseppe Craparo, Roberta Messina, Sergio Severino

**Affiliations:** ^1^University of Enna "Kore"Faculty of Human and Social SciencesEnnaItaly

**Keywords:** IAT, Internet, addiction, factorial structure, psychometric properties, structural validity

## Abstract

**Background:**

The Internet Addiction Test (IAT) by Kimberly Young is one of the most utilized diagnostic instruments for Internet addiction. Although many studies have documented psychometric properties of the IAT, consensus on the optimal overall structure of the instrument has yet to emerge since previous analyses yielded markedly different factor analytic results.

**Objective:**

The objective of this study was to evaluate the psychometric properties of the Italian version of the IAT, specifically testing the factor structure stability across cultures.

**Methods:**

In order to determine the dimensional structure underlying the questionnaire, both exploratory and confirmatory factor analyses were performed. The reliability of the questionnaire was computed by the Cronbach alpha coefficient.

**Results:**

Data analyses were conducted on a sample of 485 college students (32.3%, 157/485 males and 67.7%, 328/485 females) with a mean age of 24.05 years (SD 7.3, range 17-47). Results showed 176/485 (36.3%) participants with IAT score from 40 to 69, revealing excessive Internet use, and 11/485 (1.9%) participants with IAT score from 70 to 100, suggesting significant problems because of Internet use. The IAT Italian version showed good psychometric properties, in terms of internal consistency and factorial validity. Alpha values were satisfactory for both the one-factor solution (Cronbach alpha=.91), and the two-factor solution (Cronbach alpha=.88 and Cronbach alpha=.79). The one-factor solution comprised 20 items, explaining 36.18% of the variance. The two-factor solution, accounting for 42.15% of the variance, showed 11 items loading on Factor 1 (Emotional and Cognitive Preoccupation with the Internet) and 7 items on Factor 2 (Loss of Control and Interference with Daily Life). Goodness-of-fit indexes (NNFI: Non-Normed Fit Index; CFI: Comparative Fit Index; RMSEA: Root Mean Square Error of Approximation; SRMR: Standardized Root Mean Square Residual) from confirmatory factor analyses conducted on a random half subsample of participants (n=243) were satisfactory in both factorial solutions: two-factor model (χ^2^_132_= 354.17, *P*<.001, χ^2^/df=2.68, NNFI=.99, CFI=.99, RMSEA=.02 [90% CI 0.000-0.038], and SRMR=.07), and one-factor model (χ^2^_169_=483.79, *P*<.001, χ^2^/df=2.86, NNFI=.98, CFI=.99, RMSEA=.02 [90% CI 0.000-0.039], and SRMR=.07).

**Conclusions:**

Our study was aimed at determining the most parsimonious and veridical representation of the structure of Internet addiction as measured by the IAT. Based on our findings, support was provided for both single and two-factor models, with slightly strong support for the bidimensionality of the instrument. Given the inconsistency of the factor analytic literature of the IAT, researchers should exercise caution when using the instrument, dividing the scale into factors or subscales. Additional research examining the cross-cultural stability of factor solutions is still needed.

## Introduction

### Overview

The current overview of global Internet usage provides a striking picture of the extent of the phenomenon. Because of a steady strengthening between computer technology and traditional communication processes [1-3], Internet users’ growth reached 566.4% from 2000 to 2012. Because the majority of online users have become members of chats, forums, and social networks, the rise and popularity of the Internet is strongly linked to its use in communication and socialization processes. For this, the medium has become an ever-increasing part of many people’s day-to-day lives [4], changing the way to communicate. According to several researchers, aseptic characteristics of Computer Mediated Communication (CMC) made virtual relationships “shallow and impersonal” [3] and online anonymity produces a psychological state characterized by the abandonment of social pressures [5].

Internet addiction [4,6-8] is generally categorized under the label of “technological addiction” and is defined by Kandell as a “psychological dependence on the Internet regardless of the type of activity once logged on” [9]. Enough agreement exists on the association between Internet addiction and material and psychological consequences [10], such as the neglect of academic, work, and domestic responsibilities, disruption of relationships, social isolation, and financial problems [11]. Furthermore, literature [12-21] has amply demonstrated that pre-existing familial and social problems, as well as psychological and psychiatric disturbances are more prevalent among dependent Internet users.

### Internet Addiction Test (IAT) by Young

One of the most common diagnostic instruments for Internet addiction was proposed by Young in 1996. The author pioneered the study on Internet addiction, developing a structured Internet Addiction Test (IAT) on the basis of the DSM-IV criteria (Diagnostic and Statistical Manual of Mental Disorders, 4^th^Edition) for pathological gambling [22]. In its first version, IAT comprised eight questions and was administered to a group of subjects recruited through various announcements in newspapers, online forums, and websites. If respondents answered “yes” to five or more of the criteria, they were classified as Dependents. A total of 396 subjects fell into the Internet-Addicted user category, while 100 respondents were labelled as Non-Addicted. Most relevant results revealed that Internet-Addicted users spent approximately eight times the number of hours per week as that of Non-Dependents in using the Internet. Moreover, different from Non-Dependents (who used the medium essentially to manage email, look for information, or download software), Dependent users spent most of the time in synchronous communication environments, chat rooms, and MUDs (multi-user domains). This caused severe impairment in academic, relationship, financial, and occupational life areas.

Later, Young extended the previous version of IAT [12]. The new scale exhibits the following characteristics:

- It comprises 20 items rated in a five-point Likert scale (from 1 - not at all, to 5 - always).
- As with the first diagnostic questionnaire, this measurement is derived from the DSM–IV criteria for pathological gambling and alcoholism and it measures the extent of individual’s problems due to the Internet use in daily routine, social life, productivity, sleeping patterns, and feelings.
- On the basis of the total score obtained on the test, the individual is placed into one of three categories: average online user (from 20 to 39) who has a full control of his or her usage; experiences frequent problems because of excessive Internet use (from 40 to 69); or has significant problems because of Internet use (from 70 to 100).

Though the IAT is one of the most common instruments to assess Internet addiction, its use remains problematic. Indeed, empirical researches on Internet addiction provided conflicting results on its psychometric properties; moreover, the instrument has not been subjected to rigorous and systematic psychometric investigations [23].

Widyanto and McMurran administered the IAT on 86 subjects recruited online. The factor analysis of the IAT items revealed six factors (salience, excessive use, neglect work, anticipation, lack of control, neglect social life), with good internal consistency and concurrent validity [11]. In a more recent study, conducted on 236 Internet chatters, Ferraro, Caci, D’Amico, and Di Blasi found a six-factor solution, with an explained variance of 55.6%. The six factors were named as follows: compromised social quality of life, compromised individual quality of life, compensatory usage of the Internet, compromised academic/working careers, compromised time control, and excitatory usage of the Internet [24]. Although both surveys converge toward a six-factor solution, these factors did not correspond to the same items in the two studies [25]. Furthermore, Barke, Nyenhuis, and Kröner-Herwig administered the German version of the IAT in a large sample of students [26]. Factor analysis revealed a stable two-factor solution: Factor 1, “Emotional and Cognitive Preoccupation with the Internet”, which explained 21.03% of the variance for the offline sample and 26.73% for the online sample, and Factor 2, “Loss of Control and Interference with Daily Life”, which explained 20.97% of the variance for the offline sample and 19.99% of the variance for the online sample. The first factor encompasses items on the emotional and cognitive elements related to use of Internet. The second factor is composed of items on “(unsuccessful) attempts at curbing online time and detrimental consequences of the Internet use for daily functioning” (p. 541 [26]). The two-factor solution fit well with data also in a study conducted by Watters, Keefer, Kloosterman, Summerfeldt, and Parker in a large sample of Canadian high-school students [27]. Finally, exploratory and confirmatory factor analysis applied on the Arabic [28] and French [25] versions of the IAT revealed that a one-factor model fits the data very well.

The heterogeneity of these results could be attributed to several causes, such as the fact that many studies have used this scale in various settings [29], focusing on subjects of different ages and nationalities.

The aim of the present study is to provide a contribution in assessing the psychometric properties of the IAT in a sample of Italian college students, specifically testing its factor structure stability across cultures.

## Methods

### Participants and Procedure

Of the 521 Italian adults screened, 36 had one or more items with missing values and were not included in data analyses. Thus, participants totalled 485 (32.3%, 157/485 males and 67.7%, 328/485 females) with a mean age of 24.05 years (SD 7.3, range 17-47). The group of participants were recruited on a voluntary basis.

Confirmatory factor analyses were performed on a random subsample (sample 2) of 243 participants (35.8%, 87/243 male and 64.2%, 156/243 female), ranging in age from 18 to 50 years (mean 22.12, SD 5.9).

### Data Analyses

In order to determine the dimensional structure underlying the questionnaire, data from the 485 participants were subjected to exploratory factor analysis. With the 20-item questionnaire, we were able to satisfy the minimum 10 participants-per-item ratio that is usually recommended; a number of 24.25 subjects per item largely ensured that reliable factors would emerge.

Prior to exploratory factor analysis, data were inspected to ensure items were significantly correlated, using Bartlett’s Test of Sphericity. Also, in order to evaluate whether items share sufficient variance to justify factor extraction, KMO’s Test of Sampling Adequacy was used. Sampling adequacy values greater than .80 and .90 are considered excellent, values between .50 and .60 marginally acceptable, and values less than .50 unacceptable [30].

Principal axis factoring with oblique rotation (promax criterion) was selected as the method of factor extraction. To determine the number of factors, both Kaiser’s [31] criterion (items with eigenvalues greater than 1) and the Scree test [32] were used. Random data parallel analysis [33] was also performed. The eigenvalues derived from the actual data were compared to the eigenvalues derived from the random data. Factors were retained as long as the *i*th eigenvalue from the actual data was greater than the *i*th eigenvalue from the random data [34].

The reliability of the questionnaire, in terms of internal consistency, was computed by the Cronbach alpha coefficient. Corrected item-scale correlations were examined assuring they exceeded .30, recommended as the standard for supporting internal consistency [35].

The IAT factor structure that emerged from exploratory factor analysis was verified using the structural equation modelling technique. In particular, a confirmatory factor analysis was conducted on the data from the random subsample of participants (sample 2). Least Square, which is applicable when data do not meet the assumption of multivariate normality, was selected as the procedure for estimation.

The closeness of the hypothetical model to the empirical data was statistically evaluated through multiple goodness-of-fit indexes. Chi-square is sensitive to sample size and may be significant when the actual differences between the observed and implied model covariances are slight [36]. Therefore, we did not use this statistic as an evaluation of absolute fit, but referred to the ratio of chi-square to degrees of freedom (χ^2^/df [37]), the Non-Normed Fit Index (NNFI [38]), the Comparative Fit Index (CFI [39]), and the Standardized Root Mean Square Residual (SRMR [39]) to evaluate adequacy of fit of each model. We also reported the Root Mean Square Error of Approximation (RMSEA [40]) to provide an indication of the global fit of the model. Model testing was accomplished using the EQS (version 6.1) structural equations modeling software package [41]. Higher values for the CFI and NNFI are considered good (>.90, acceptable and >.95, desirable [42]). The RMSEA is an index of misfit per degree of freedom; lower values are preferred (<.08, acceptable, <.05, desirable [42]). The SRMR is the average standardized deviation in the model-based reproduced covariances in contrast to those observed in the data; lower values are optimal (<.10, acceptable, <.05, desirable [42]).

## Results

### Participants

A series of analyses was conducted to examine the psychometric properties of the questionnaire, including reliability and both exploratory and confirmatory factor analyses. Results showed 176/485 (36.3%) participants with IAT score from 40 to 69, revealing excessive Internet use, and 11/485 (1.9%) participants with IAT score from 70 to 100, suggesting significant problems because of Internet use.

### Exploratory Factor Analysis

The KMO’s Test of Sampling Adequacy was .94 and Bartlett’s Test of Sphericity (χ^2^_190_=4014.0) was significant (*P*<.001), indicating that the IAT items were appropriate for a factor analysis.

We employed Horn’s [33] parallel analysis (PA) for determining the number of factors to retain because it has been shown empirically to give accurate results [43]. This criterion involves comparison of eigenvalues for data under study with those extracted from and averaged over a large number of random data sets (we used 1000) based on the same number of variables and subjects. If eigenvalue I for data under study exceeds the average over a large number of random data-based eigenvalues I, that factor is retained. One then proceeds to factor II and so on, retaining only the number of factors for which real data-based eigenvalues exceed averages derived from random data. Parallel analysis determined five factors to be extracted. The resulting number of factors is evidently over-defined, with two factors comprised by only two indicators, one item failed to load .30 or greater in any factor, and 11 items loaded simultaneously on two factors without a difference of at least .30 between loading on the primary factor and loading on other factors.

As a consequence of these poor findings, we followed the eingenvalues-greater-than-one criterion, extracting three factors but rotation (both orthogonal and oblique) failed to converge. Examination of the scree plot suggested two factors to be extracted. Inspection of factor loadings revealed 18 items to have been appropriate, having pattern coefficients of .35 or greater, which is generally regarded as the standard for pattern coefficient cutoff criteria [44]. Item 17 (“Do you try to cut down the amount of time you spend online and fail?”) and item 8 (“Does your job performance or productivity suffer because of the Internet?”) presented double loadings and were eliminated. The two-factor solution, accounting for 42.15% of the variance, showed 11 items loading on Factor 1 (Emotional and Cognitive Preoccupation with the Internet), and 7 items on Factor 2 (Loss of Control and Interference with Daily Life); we utilized the same wording proposed by Barke, Nyenhuis, and Kröner-Herwig [26]. Table 1 depicts the pattern coefficients for the two-factor solution. Factors intercorrelation according to the results of exploratory factor analysis was .65. Correlations between the two-factor mean scores (ie, sum of the items/number of items) was .64 (*P*<.01). To be thorough, an exploratory factor analysis requesting one factor was also performed. The eigenvalue and variance accounted for the factor were 7.24 and 36.18%, respectively. Table 2 reports factor loadings of the IAT item for the one-factor solution.

### Reliability

The reliability of the IAT was assessed for both one- and two-factor structure models. Internal consistency was assessed with coefficient alpha for the entire sample of 485 participants. Satisfactory results were evident for both one-factor solution (Cronbach alpha=.91, see Table 2) and two-factor solution (Factor 1 Cronbach alpha=.88 and Factor 2 Cronbach alpha=.79; see Table 3).

**Table 1:** Factor loadings of the IAT items for the two-factor solution.

Items^a^	Factor 1^b^	Factor 2^c^
20. Do you feel depressed, moody, or nervous when you are offline, which goes away once you are back online?	.940	
15. Do you feel preoccupied with the Internet when offline or fantasize about being online?	.694	
3. Do you prefer the excitement of the Internet to intimacy with your partner?	.678	
19. Do you choose to spend more time online over going out with others?	.649	
18. Do you try to hide how long you’ve been online?	.628	
11. Do you find yourself anticipating when you go online again?	.623	
12. Do you feel that life without the Internet would be boring, empty, and joyless?	.622	
13. Do you snap, yell, or act annoyed if someone bothers you while you are online?	.518	
10. Do you block disturbing thoughts about your life with soothing thoughts of the Internet?	.473	
4. Do you form new relationships with fellow online users?	.443	
14. Do you lose sleep due to late night log-ins?	.414	
2. Do you neglect household chores to spend more time online?		.803
1. Do you feel that you stay online longer than you intend?		.761
16. Do you find yourself saying “just a few more minutes” when online?		.595
6. Does your work suffer because of the amount of time you spend online?		.549
5. Do others in your life complain to you about the amount of time you spend online?		.542
9. Do you become defensive or secretive when someone asks what you do online?		.403
7. Do you check your email before something else that you need to do?		.372
% explained variance	36.08	6.07

^a^Items are ordered by factor loading rather than item number.

^b^Factor 1: Emotional and Cognitive Preoccupation with the Internet

^c^Factor 2: Loss of Control and Interference with Daily Life

**Table 2:** Factor loadings of the IAT items and corrected item-total correlations for the one-factor solution.

Items^a^	Loadings	Item-total correlations
11. Do you find yourself anticipating when you go online again?	.705	.670
15. Do you feel preoccupied with the Internet when offline or fantasize about being online?	.699	.647
5. Do others in your life complain to you about the amount of time you spend online?	.687	.666
6. Does your work suffer because of the amount of time you spend online?	.680	.656
13. Do you snap, yell, or act annoyed if someone bothers you while you are online?	.674	.640
18. Do you try to hide how long you’ve been online?	.664	.621
20. Do you feel depressed, moody, or nervous when you are offline, which goes away once you are back online?	.662	.606
8. Does your job performance or productivity suffer because of the Internet?	.656	.622
19. Do you choose to spend more time online over going out with others?	.646	.603
10. Do you block disturbing thoughts about your life with soothing thoughts of the Internet?	.636	.606
14. Do you lose sleep due to late night log-ins?	.611	.573
17. Do you try to cut down the amount of time you spend online and fail?	.610	.581
12. Do you feel that life without the Internet would be boring, empty, and joyless?	.597	.558
16. Do you find yourself saying “just a few more minutes” when online?	.589	.577
2. Do you neglect household chores to spend more time online?	.550	.548
9. Do you become defensive or secretive when someone asks what you do online?	.529	.517
4. Do you form new relationships with fellow online users?	.486	.461
3. Do you prefer the excitement of the Internet to intimacy with your partner?	.450	.401
1. Do you feel that you stay online longer than you intend?	.417	.424
7. Do you check your email before something else that you need to do?	.300	.295
% explained variance	36.18	
Cronbach alpha		.91

^a^Items are ordered by factor loading rather than item number.

**Table 3:** Corrected item-total correlations.

Item^a^	Factor 1^b^	Factor 2^c^
Item 20	.708	
Item 15	.668	
Item 3	.491	
Item 19	.631	
Item 18	.616	
Item 11	.692	
Item 12	.595	
Item 13	.627	
Item 10	.588	
Item 4	.467	
Item 14	.535	
Item 2		.603
Item 1		.520
Item 16		.550
Item 6		.603
Item 5		.619
Item 9		.472
Item 7		.325
Cronbach alpha	.88	.79

^a^Items are ordered by factor rather than item number.

^b^Factor 1: Emotional and Cognitive Preoccupation with the Internet

^c^Factor 2: Loss of Control and Interference with Daily Life

### Confirmatory Factor Analysis

**Figure 1:**
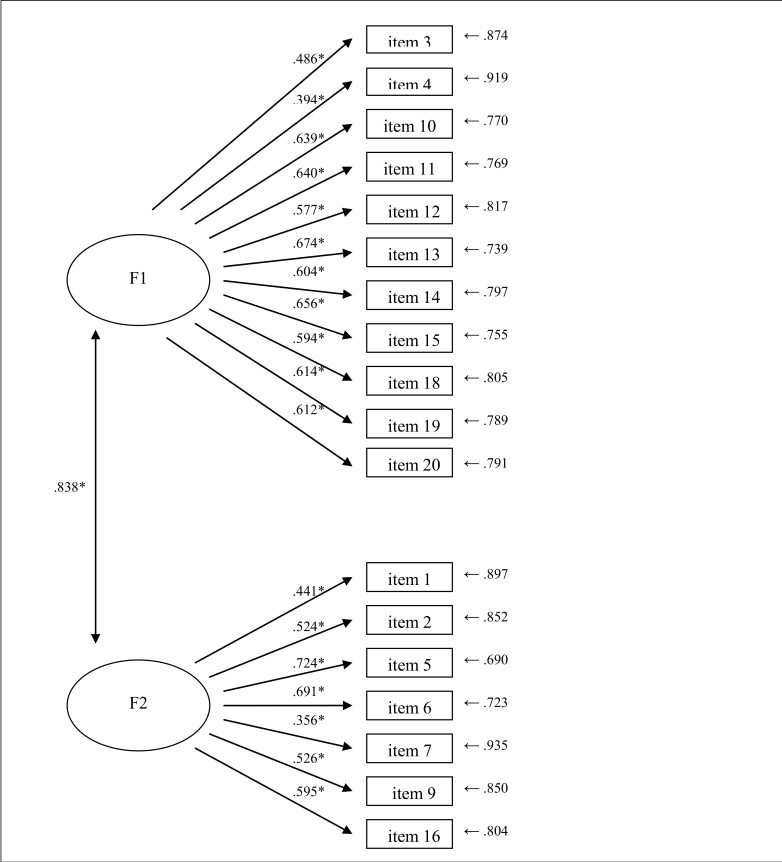
IAT empirical model (standardized solution). Note: F1 = Emotional and Cognitive Preoccupation with the Internet; F2 = Loss of Control and Interference with Daily Life. * P<.05.

The confirmatory factor analyses (CFA) conducted on sample 2 (n=243) showed the acceptable goodness-of-fit indexes for the two-factor model (χ^2^_132_=354.17; *P*<.001, χ^2^/df=2.68, NNFI=.99, CFI=.99, RMSEA=.02 [90% CI 0.000-0.038], and SRMR=.07). All manifest variables loaded significantly (*P*<.05) on their hypothesized latent factors. Figure 1 shows the standardized parameter estimates.

According to the results of the CFA, the latent factors are highly correlated to each other. Specifically, they share 70.22% of common variance indicating poor discriminant validity between extracted factors and maybe a more parsimonious solution could be obtained.

Consequently, confirmatory analysis was performed on all IAT items to test for unidimensionality. The completely standardized factor loadings are reported in Table 4. Table 5 contains results for both two-factor and one-factor models specified and tested.

The comparative fit of the models was assessed with the Akaike Information Criterion (AIC [45,46]), which is used for model comparison, with the smallest value being indicative of the best fitting model. AIC for the one-factor model was 145.79, AIC for the two-factor model was 90.17, providing greater support for the bidimensionality of the instrument.

**Table 4:** Standardized factor loadings of the IAT items for the one-factor solution.

Items	Loadings	Residuals
1. Do you feel that you stay online longer than you intend?	.406	.914
2. Do you neglect household chores to spend more time online?	.484	.875
3. Do you prefer the excitement of the Internet to intimacy with your partner?	.475	.880
4. Do you form new relationships with fellow online users?	.377	.926
5. Do others in your life complain to you about the amount of time you spend online?	.675	.738
6. Does your work suffer because of the amount of time you spend online?	.668	.745
7. Do you check your email before something else that you need to do?	.347	.938
8. Does your job performance or productivity suffer because of the Internet?	.670	.742
9. Do you become defensive or secretive when someone asks what you do online?	.507	.862
10. Do you block disturbing thoughts about your life with soothing thoughts of the Internet?	.618	.786
11. Do you find yourself anticipating when you go online again?	.610	.793
12. Do you feel that life without the Internet would be boring, empty, and joyless?	.546	.838
13. Do you snap, yell, or act annoyed if someone bothers you while you are online?	.633	.774
14. Do you lose sleep due to late night log-ins?	.584	.812
15. Do you feel preoccupied with the Internet when offline or fantasize about being online?	.650	.760
16. Do you find yourself saying “just a few more minutes” when online?	.563	.827
17. Do you try to cut down the amount of time you spend online and fail?	.582	.813
18. Do you try to hide how long you’ve been online?	.586	.810
19. Do you choose to spend more time online over going out with others?	.586	.810
20. Do you feel depressed, moody, or nervous when you are offline, which goes away once you are back online?	.594	.804

**Table 5:** Fit indices for the one-factor and two-factor models.

Model	χ^2^	df	*P* value	NFI^a^	NNFI^b^	CFI^c^	SRMR^d^	RMSEA^e^	90% CI
One-factor model	483.79	169	<.001	.895	.984	.986	.070	.024	0.000-0.039
Two-factor model	354.17	132	<.001	.906	.989	.991	.067	.020	0.000-0.038

^a^NFI: Normed Fit Index

^b^NNFI: Non-Normed Fit Index

^c^CFI: Comparative Fit Index

^d^SRMR: Standardized Root Mean Square Residual

^e^RMSEA: Root Mean Square Error of Approximation

## Discussion

### Principal Findings

The present study examined the model of Internet addiction as assessed by a widely used self-report measure, the IAT. In line with many previous studies suggesting the need to test the factor structure stability across cultures and samples of commonly used instruments in several fields of psychological research [47-52], we sought to document the factor structure of the scale, with the final aim to enhance our understanding of the Internet addiction construct.

Knowledge of the structure of the IAT and its consistency over cultures and languages can serve a number of useful purposes: advance theory regarding the place of the disorder within the nosology of psychiatric conditions, hence contributing to the development of accurate and valid assessment tools.

Extant research on the factor structure of IAT has done much to highlight key issues in the dimensionality of the construct, yet several concerns warrant further empirical attention. Indeed, although it remains one of the most broadly used measures of Internet addiction worldwide, its factor structure remains questionable. Thus, factor analytic research on the IAT is important for the psychometric evaluation of the instrument and for clarifying the nature of the Internet addiction construct itself.

Many studies have documented psychometric properties of the IAT, with markedly different factor analytic results. Consensus on the optimal overall structure has yet to emerge since previous analyses have found between one- and six-factor solutions for the IAT.

Our study was aimed at determining the most parsimonious and veridical representation of the structure of Internet addiction as measured by the IAT. Based on our findings, support was provided for both single- and two-factor models (Factor 1: Emotional and Cognitive Preoccupation with the Internet; Factor 2: Loss of Control and Interference with Daily Life) with slightly strong support for the bidimensionality of the instrument. Nevertheless, the two-factor solution presents some limitations due to the resulting high association between emerged factors. Indeed, different dimensions are generally expected not to be highly correlated, indicating that the subscales measure several aspects of the investigated construct. However, the revealed high associations between factors is understandable because of the unavoidable conceptual connection of the questionnaires’ subscales, also found in previous studies [26]. Otherwise, the more parsimonious solution, though usable, would be less effective for a detailed assessment of Internet addiction with consequential loss of salient information.

### Limitations

Overall, our findings should be interpreted with some caution because the sample contained only college students. This condition is tempered by the fact that they are an at-risk population in which intense Internet use is common and potentially consequential [9,53]. Clearly, more research needs to be conducted with larger and more varied samples of participants to further establish the structural validity of the instrument.

### Conclusions

In summary and in closing, on the basis of the present results combined with inconsistency of the factor analytic literature of the IAT, it seems apparent that researchers should be aware of these psychometric issues and exercise caution when using the IAT, dividing the scale into factors or subscales. Preliminary evidence of scale validity is encouraging; however, additional research examining the cross-cultural stability of factor solutions is still needed.

## Abbreviations

AIC: Akaike Information Criterion
CFA: Confirmatory Factor Analysis
CFI: Comparative Fit Index
CMC: Computer Mediated Communication
IAT: Internet Addiction Test
NFI: Normed Fit Index
NNFI: Non-Normed Fit Index
RMSEA: Root Mean Square Error of Approximation
SRMR: Standardized Root Mean Square Residual
